# Post hoc analysis of a randomized controlled trial comparing concurrent chemoradiation with cisplatin versus nimotuzumab-cisplatin, focusing on acute oral mucositis

**DOI:** 10.1186/s43046-021-00069-1

**Published:** 2021-05-22

**Authors:** Vanita Noronha, Vijay M. Patil, Gunjesh Kumar Singh, Amit Joshi, Nandini Menon, Sarbani Ghosh Lashkar, Vijayalakshmi Mathrudev, Kavita Nawale Satam, Kumar Prabhash

**Affiliations:** 1 Department of Medical Oncology, Tata Memorial Hospital, Mumbai, 400012 India; 2 Department of Radiotherapy, Tata Memorial Hospital, Mumbai, 400012 India

**Keywords:** Acute oral mucositis, Head and neck cancer, Chemoradiation

## Abstract

**Background:**

Acute oral mucositis has been infrequently studied in the patients with head and neck squamous cell carcinoma (HNSCC) receiving once-weekly cisplatin-based chemoradiotherapy (CRT). Hence, this analysis was conducted to explore the various aspects of the same.

**Results:**

The overall incidence of mucositis was 96.9% (*n* = 508) and of grade 3–5 mucositis was 61.3% (*n* = 321). The overall incidence of oral mucositis was similar in both the arms (CCRT and NCRT) (*p* value = 0.58) while grade 3–5 mucositis was more common in the NCRT arm (*p* value = 0.01). Out of all factors listed, the presence of nimotuzumab was the only significant risk factor for the development of grade 3 or more oral mucositis (*p* value = 0.01); (OR = 1.64, 95%CI 1.15–2.32). Delays in the treatment delivery were similar in both the arms.

**Conclusion:**

Acute oral mucositis is a common occurrence in locally advanced-HNSCC patients receiving chemoradiotherapy. Nimotuzumab is a significant factor for development of grade 3 and above oral mucositis.

## Background

Cisplatin-based chemoradiotherapy (CRT) has been the standard treatment for patients with locally advanced head and neck squamous cell carcinomas (LA-HNSCC), given with the aim of preserving function and improving survival [1]. However, this intensive chemoradiotherapy is associated with significant acute and late toxicities [2]. The data with regard to the acute adverse events especially oral mucositis with 100 mg/m^2^ of 3-weekly CRT is well documented; however, there is a dearth of data in case of weekly CRT regime. While comparing 3-weekly and once weekly CRT regimes in a retrospective study, Tsan et al. found a higher rate of oral mucositis and overall toxicity in the latter [3]. In a meta-analysis by Szturz et al, higher dose cisplatin given three to four weekly was found less toxic when compared to low dose cisplatin (< 50 mg/m^2^) given once weekly [4]. Oral mucositis can also complicate oral candidiasis which is commonly observed in these patients and increase susceptibility to fungemia [5]. High-grade oral mucositis and their complications often lead to treatment delay resulting in poor survival outcomes.

In our institution, weekly CRT with cisplatin at a dose of 30 mg/m^2^ is being used for chemoradiotherapy for 14 years (2006). Theoretically, once weekly CRT appears better tolerable but there is literary evidence of it being rather more toxic. There is no information till date regarding the pattern of oral mucositis, its presentation, peak, and recovery in patients who are given once weekly CRT. Patients with dysphagia due to tumor proximity and those with their swallowing apparatus in the radiotherapy treatment field are at high risk for development of toxicities. Hence, it becomes exceedingly important to keep these patients (HNSCC on chemoradiotherapy) well informed about the time and extent of occurrence of mucositis and the related complications possible during the treatment.

The purpose of this study was to evaluate the peak incidence, severity, and the pattern of presentation of oral mucositis along with the factors affecting it in a cohort of patients with HNSCC undergoing concurrent chemoradiotherapy. The knowledge will help in patient education on the treatment and its effects and will also guide clinical support services.

## Methods

### Study details

This was a retrospective post hoc analysis of a prospective randomized controlled trial comparing weekly concurrent cisplatin (30 mg/m^2^) (CCRT) with weekly Nimotuzumab-CRT (NCRT) from 2012 to 2018 [6]. Approval for this study was obtained from the Institutional Review Board. A written informed consent was obtained from every patient before participation. Newly diagnosed treatment-naive adult patients with non-metastatic, stage III or IV LA-HNSCC arising in the oropharynx, larynx, hypopharynx, or oral cavity were eligible for the study. The other criteria were a Karnofsky performance status ≥ 70 and adequate hematological, renal, and hepatic functions. Patients with tumors originating in the nasopharynx, salivary gland, or nasal cavity and those who had received immunotherapy or prior radiotherapy to the head and neck region were excluded. In both the arms, cisplatin was dosed at 30 mg/m^2^ weekly along with radiation therapy and supportive medications. Nimotuzumab was administered weekly in the NCRT arm intravenously as a 200-mg flat dose in 250 mL normal saline over 60 min without any premedication. Patients received prior hydration, 5HT3 antagonists along with dexamethasone and/or neurokinin-1 receptor antagonist as antiemetic prophylaxis. All the patients were also provided weekly oral care by the dentists in addition to proper education on skin care by pharmacists and nurses. The toxicities were graded using the Common Terminology Criteria for Adverse Events (NCI-CTCAE) Version 4.03 [7].

### Data collection

Data related to oral mucositis during the course of chemoradiotherapy, i.e., from week 0 to 7 of treatment was collected. The information on the occurrence of other acute toxicities was also captured and collected along with chemotherapy (CCRT and NCRT) delivery details. The entire data was entered in an excel sheet and the following details were noted:

1. Incidence, severity of oral mucositis in both CCRT and NCRT arm.
2. Factors affecting acute oral mucositis—age (older, i.e., ≥ 60 years vs younger), gender, Eastern Cooperative Oncology Group performance score (ECOG-PS), stage of primary, site of primary, the technique of radiotherapy used, and chemotherapy regimen (weekly CCRT and NCRT).
3. The severity of other acute adverse events such as rise in serum creatinine level, odynophagia, dysphagia, weight loss, and dermatitis were also noted. Purpose of this detailed data collection was to see their association with oral mucositis.
4. The impact of oral mucositis on treatment delay and hospitalization rates due to severe oral mucositis was also studied.

### Statistical analysis

The incidence rates of acute mucositis were computed and the factors affecting it were sought, following which, the cumulative incidence rate was calculated using a competing risk analysis. SPSS version 20 was used for statistical analysis. Grays test was used to compare the incidence rates between nimotuzumab and non-nimotuzumab regimens. To see the relationship between mucositis and various factors, Fisher test (2-sided) and binary logistic regression analysis were used for univariate analysis. The relationship between complications and mucositis was studied by using fisher test. *p* value ≤ 0.05 was considered significant.

## Results

### Incidence of mucositis

Out of 536 patients, acute adverse events were captured in 524 patients. The overall incidence of mucositis was 96.9% (*n* = 508) while for grade 3–5 mucositis it was 61.3% (*n* = 321). Out of these 524 patients, 260 were in the CCRT group and 264 in the NCRT group. The overall incidence of oral mucositis was 96.9% (*n* = 252) while it was 55.8% (*n* = 145) for grade 3–5 in the CCRT group. In comparison, in the NCRT group, the overall incidence was 97% (*n* = 256) (*p* value = 0.58) while that of grade 3–5 mucositis was 66.7% (*n* = 176) (*p* value = 0.01).

**Fig. 1:**
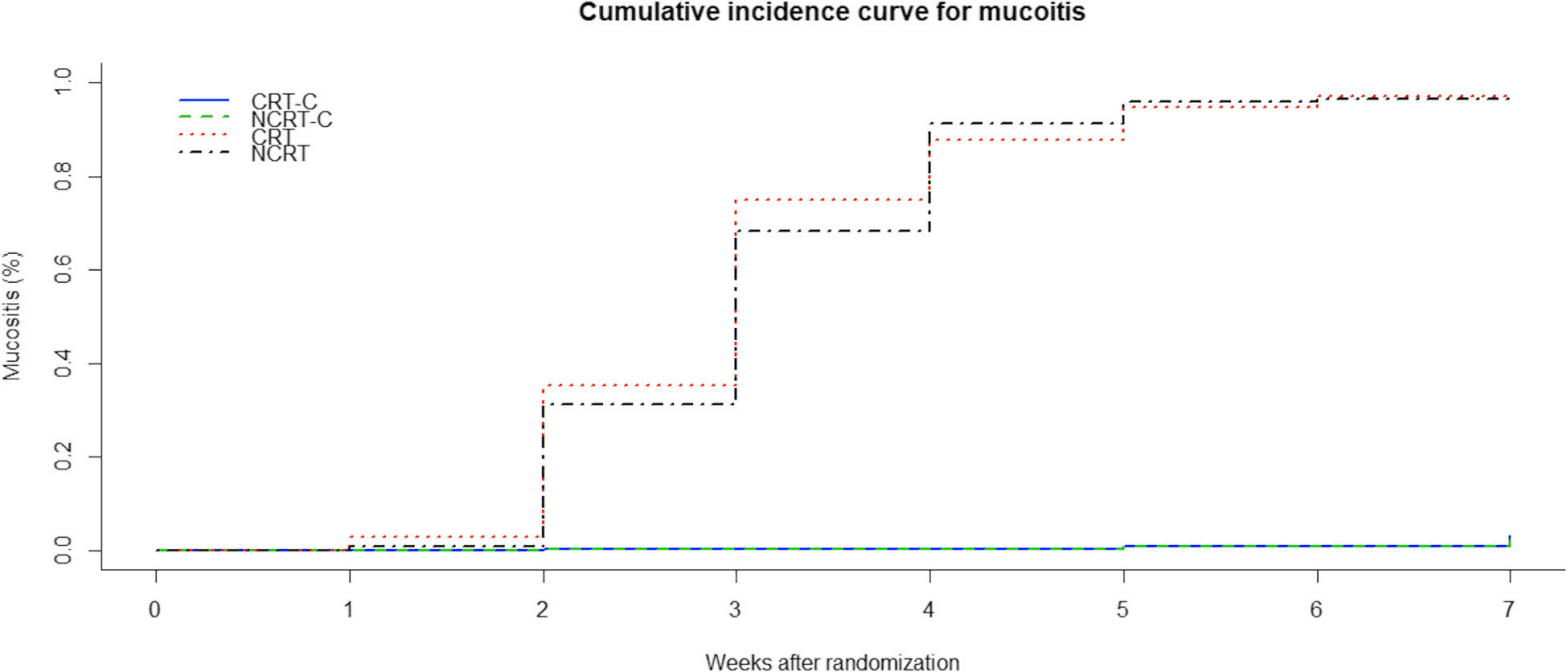
Cumulative incidence of mucositis

**Fig 2:**
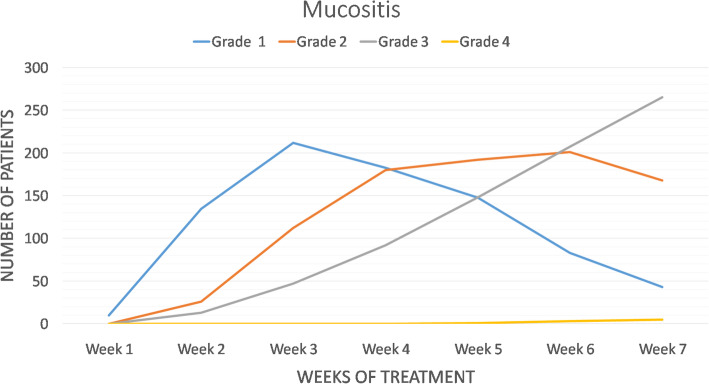
Temporal evolution of the pattern of mucositis per week

**Fig. 3:**
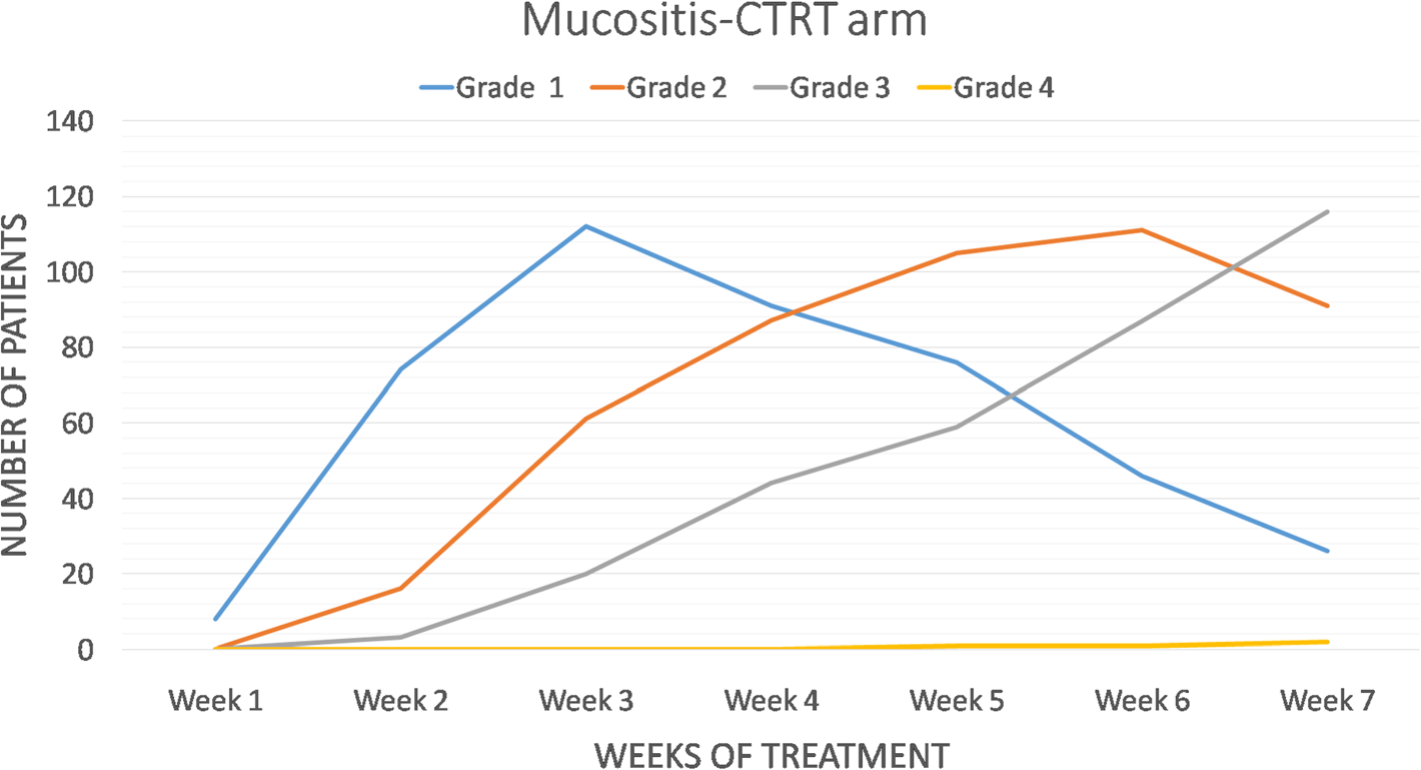
Temporal evolution of the pattern of mucositis per week in cisplatin arm

**Fig. 4:**
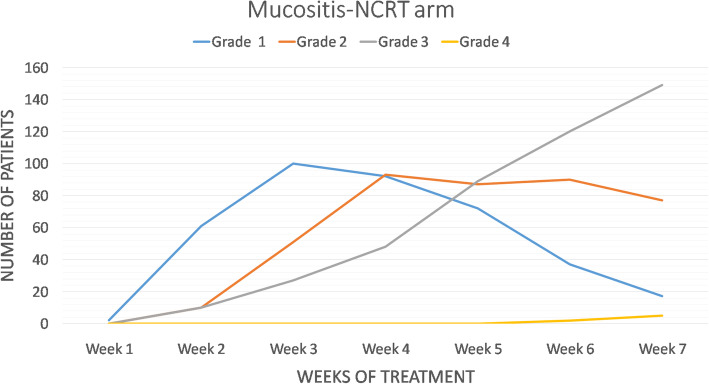
Temporal evolution of the pattern of mucositis per week in nimotuzumab cisplatin arm

The cumulative incidence of mucositis in both the arms is shown in Fig. 1. The temporal evolution of the pattern of mucositis per week of all patients and patients in each arm according to the treatment regimen is shown in Figs. 2, 3, and 4. The median time to develop any grade mucositis was 3 weeks (95%CI 2.9–3.1) while it was 7 weeks (95%CI 6.7–7.2) for grade 3–5 mucositis.

### Factors associated with mucositis

We found that none of the listed factors was associated with an increased risk of development of any grade mucositis (Table 1). However, the presence of nimotuzumab in the NCRT regime did add to the risk of grade 3 or more mucositis (*p* value = 0.01); (OR 1.64, 95%CI 1.15–2.32) (Table 2).

**Table 1:** Factors affecting any grade oral mucositis

	Number of patients (*n*)	Any grade mucositis (*n*, %)	Univariate *p* value
Arm			
Cisplatin	260	252 (96.9%)	1.000
Cisplatin-nimotuzumab	264	256 (96.9%)	
Gender			
Male	445	431 (96.9%)	1
Female	79	77 (97.5%)	
ECOG PS			
PS 0	116	115 (99.1%)	0.216
PS 1–2	408	393 (96.3%)	
Stage			
III	163	158 (96.9%)	1
IV	361	350 (96.9%)	
Radiotherapy technique			
Conventional	458	443 (96.7%)	0.706
IMRT	66	65 (98.5%)	
Age (continuous variable)			
Age	536	–	–

*ECOG-PS* Eastern Cooperative Oncology Group performance score, *IMRT* intensity-modulated radiation therapy

**Table 2:** Factors affecting grade 3–4 oral mucositis

	Number of patients (*n*)	Grade 3–4 mucositis (*n*, %)	Univariate *p* value
Arm			
Cisplatin	260	145 (55.8%)	0.012
Cisplatin-nimotuzumab	264	176 (66.7%)	
Gender			
Male	445	275 (61.8%)	0.616
Female	79	46 (58.2%)	
ECOG PS			
PS 0	116	68 (58.6%)	0.519
PS 1–2	408	253 (62%)	
Stage			
III	163	98 (60.1%)	0.771
IV	361	223 (61.8%)	
Radiotherapy technique			
Conventional	458	279 (60.9%)	0.787
IMRT	66	42 (63.6%)	
Age (continuous variable)			
Age	536	–	–

*ECOG-PS* Eastern Cooperative Oncology Group performance score, *IMRT* intensity-modulated radiation therapy

### Impact of mucositis on treatment delivery

The presence of oral mucositis delayed chemotherapy for 3 or more days in 32.5% (*n* = 87) and 30.6% (*n* = 82) patients in the CCRT and NCRT arms respectively (*p* value = 0.642). Again, patients who required chemotherapy dose modification due to mucositis were 7.8% (*n* = 21) and 9.7% (*n* = 26) in the CCRT and NCRT arm respectively (*p* value = 0.445). Patients who received cumulative chemotherapy with < 7 cycles of weekly cisplatin due to mucositis were 12.7% (*n* = 34) and 13.1% (*n* = 35) in the CCRT and NCRT arm respectively (*p* value = 0.445). Patients who had a delay of radiotherapy for 3 or more days were 3.7% (*n* = 10) and 4.5% (*n* = 12) in CCRT and NCRT arm (*p* value = 0.642), respectively.

### Association of oral mucositis with other toxicities

We observed that other toxicities such as dysphagia, weight loss, and dermatitis of any grade were also associated with oral mucositis and this association was mostly seen with higher grades (grade 3 and above) of the former (Table 3). Out of all patients with acute adverse events, the ones with any grade of oral mucositis and required admission and indoor care anytime during the course of treatment were 114 (21.8%). 82 (25.5%) out of all patients with grade 3 or above oral mucositis (*n* = 321) needed admission for supportive care. Further, the hospitalization rates were also not different between patients with any grade and grade 3 or above oral mucositis (*p* value = 0.541).

**Table 3:** Association of acute oral mucositis with other toxicities

Adverse event	Grade of adverse event	Mucositis		***p*** value
		Grade 0–2 (*n =* 203)	Grade3-5 (*n =* 321)	
Rise in serum creatinine	Any grade	17 (8.4%)	32(10%)	0.645
	Grade 3–5	–	2(0.6%)	0.524
Odynophagia	Any grade	192(94.6%)	317(98.8%)	0.007
	Grade 3–5	38(18.7%)	169(52.6%)	< 0.0001
Dysphagia	Any grade	160(78.8%)	295(91.9%)	< 0.0001
	Grade 3–5	33(16.3%)	122(38.0%)	< 0.0001
Weight loss	Any grade	50(24.6%)	243(75.7%)	< 0.0001
	Grade 3–5	0(0)	5(1.6%)	0.162
Dermatitis	Any grade	168(82.8%)	304(94.7%)	< 0.0001
	Grade 3–5	38(18.7%)	111(34.6%)	< 0.0001

## Discussion

Different studies have been conducted to evaluate the frequency and possible risk factors of concurrent chemoradiotherapy (CTRT) induced oral mucositis. However, the details of the mechanism of oral mucositis development are completely unknown, and its control during CTRT remains challenging [8]. To the best of our knowledge, this is the first longitudinal study to investigate the association between oral mucositis and different risk factors during CTRT. The overall incidence of mucositis in our patients was 96.9% (508/524) and grade 3 and above were seen in 61.3% (321/524) patients. The overall incidence of oral mucositis was similar in both the arms (*p* value = 0.58); however, that of grade 3–5 was more in the NCRT arm (*p* value = 0.01). We also found that the rate of hospitalization increased with increasing severity of mucositis and 25.5% (82) patients with grade 3 or more mucositis required indoor care.

In the landmark studies by Bernier et al. and Cooper et al., they noted that cumulative grade 3 or above mucosal adverse events were more in the CCRT arm (cisplatin 100 mg/m^2^, 3-weekly) in comparison to the radiation alone arm that is 41% vs. 21%; *p* = 0.001 and 44.5% vs. 21.3%, *p* < 0.001, respectively [9, 10]. Many studies like these in the literature have described and reported the occurrence of acute oral mucositis with 3-weekly cisplatin, but there only a handful of them studying the same with the weekly cisplatin regime. Tsan et al. compared weekly and 3-weekly cisplatin regimes in a small cohort of 55 patients and found that 22 (91.7%) patients in the former group (*n* = 24) had grade 3 and above oral mucositis [3]. Noronha et al., in a randomized phase III controlled trial from India, found a similar rate of severe acute oral mucositis (grade 3 or above) in once weekly and once 3-weekly cisplatin regimens (17.3% vs 18.1%, *p* value = 0.9) [11]. Further, a systematic review of literature and meta-analysis by Szturz et al. showed that patients in the weekly cisplatin arm experienced a higher rate of grade 3 or above acute oral mucositis (75% vs 40%, *p* = .0202) [4]. Similarly, the prevalence and severity of acute oral mucositis in our study were high (61.3%).

Radiotherapy induced mucositis begins in the 2nd or 3rd week of treatment and reaches its peak at around 5th to 6th week [12]. Similarly, in our study the median time to oral mucositis was 3 weeks and it peaked at 7th week.

Along with the type of chemoradiotherapy, a variety of factors including age, nutritional status, type of malignancy, pretreatment oral condition, oral care during treatment, and pretreatment neutrophil counts are proposed to be associated with the development of acute oral mucositis in patients with HNSCC [13]. In an Indian study by Suresh et al. age > 40 years, ECOG PS > 2, total leucocyte count < 3000/μL, elevated erythrocyte sedimentation rate, serum albumin < 3 gm/dL, a primary tumor of stage 3 or more, comorbid conditions, nutritional status, oral hygiene, and tobacco use were taken as the risk factors. They used these risk factors to calculate a score. The higher the score, the more is the chance of developing acute oral mucositis [14]. Out of all risk factors listed in our study, we found that the addition of nimotuzumab was significantly linked with the development of grade 3 and above acute oral mucositis (*p* value = 0.01).

In a systematic review of literature, which included 33 studies to see the incidence, severity, and outcomes of oral mucositis, oral pain, weight loss, dysphagia, dehydration, and use of analgesics/opioid were reported as the important symptoms of oral mucositis [15]. Out of these, weight loss was the most common symptom seen in 10/33 studies. Notably, the rates of hospitalization due to acute oral mucositis were higher in patients in the chemoradiotherapy group than those who received radiotherapy alone (6% vs. 2% group) [16]. One needs to pay attention that severe oral mucositis is significantly associated with more total parenteral nutrition support and parenteral narcotic therapy. Also, there is a higher chance of infection which may further increase the duration of hospital stay and cost of inpatient supportive care [17]. In our patients, 21.8% and 25.5% patients with any grade and grade 3 and above oral mucositis required inpatient care, which was high but the actual number of patients who were admitted because of oral mucositis per se was not captured and this remains one of the limitations of our study.

It should be noted that oral mucositis can lead to treatment delay and treatment (chemotherapy and radiotherapy dose) modification. However, in both the CCRT and NCRT arms of our study, no difference in delay of chemotherapy and radiotherapy delivery was seen and cumulative doses of chemotherapy and radiotherapy were similar as well. In various studies, planned or unplanned treatment modifications have been reported frequently, but the extent or link to mucositis is rarely noted by the authors [15].

To our knowledge, this study represents the largest and most comprehensive report of acute oral mucositis and its outcomes in patients with LA-HNSCC treated with radiotherapy and concurrent chemotherapy. Overall, the current data indicates that patients continue to require supportive care for a range of treatment-related toxicities, including acute oral mucositis, particularly beginning in the first 3–4 weeks, until the final weeks of treatment with symptoms improving thereafter.

## Conclusion

This study confirms that patients with LA-HNSCC undergoing concurrent chemotherapy with radiotherapy experience a range of treatment-related toxicities including oral mucositis. Also, nimotuzumab is a significant factor associated with development of grade 3 and above oral mucositis. Hence, there is a need for contemporary evidence base to help guide the delivery of timely clinical and supportive care to these patients during treatment.

## Data Availability

The authors confirm that the data supporting the findings of this study are available within the article.

## Abbreviations

HNSCC: Head and neck squamous cell carcinoma
CRT: Cisplatin-based chemoradiotherapy
NCRT: Weekly nimotuzumab-cisplatin-based chemoradiotherapy
LA-HNSCC: Locally advanced head and neck squamous cell carcinomas
CCRT: Weekly concurrent cisplatin (30 mg/m^2^)
NCI-CTCAE: Common Terminology Criteria for Adverse Events
ECOG-PS: Eastern Cooperative Oncology Group performance score
CTRT: Concurrent chemoradiotherapy
